# Improving Clinical Communication With the Doctor On-Call: A QI Project

**DOI:** 10.1192/bjo.2024.449

**Published:** 2024-08-01

**Authors:** Henry Verrall

**Affiliations:** Sussex Partnership Foundation Trust, Brighton, United Kingdom

## Abstract

**Aims:**

Clear, accurate and efficient clinical communication between wards and on-call doctors is vital for good patient care. Issues were raised locally regarding the quality and content of these calls, and a QI project devised to assess the issue and implement meaningful change.

**Methods:**

An initial QI Audit was undertaken, using Likert scale questionnaires to rank areas of concern. These were sent to all the doctors currently manning the on-call rota, and doctors who had previously covered these on-calls. Responses were used to gauge the key concerns, and a blank space and multiple choice question on possible contributors to the issues were included.

A communication prompt was designed that tackled the key issues highlighted by the audit. A clear flow-chart ensured that safe and sensible steps were taken to maximise the efficiency of a necessary call. A summary of the SBAR communication tool was also included to encourage structured handover. These prompts were cheap and easily affixed to ward telephones and were laminated and wipe-clean. Implementation was agreed with and supported by the senior nursing team.

A post-QI questionnaire was then sent out one month after the intervention, getting feedback from the junior doctors covering on-call shifts in that time.

**Results:**

Questionnaire Likert scales measured either Frequency (1-Very Rarely – 5-Very Frequently) or Quality (1-Poor – 5-Excellent), and a mean of the scores was taken for each question.

The initial audit (n = 14) included all the doctors currently on the on-call rota (n = 7). Key issues raised were Average Call Quality (2.2/5), how frequently recent NEWS scores were available (2.3/5), and how frequently key clinical information was on hand during the call (1.9/5). Many trainees were made to feel uncomfortable or like they were being difficult for requesting more information (3.2/5). And calls were often noted to not be relevant (3.9/5) or were confusing/unclear (3.9/5).

A second questionnaire was completed 1 month post-intervention by the doctors working the on-call rota in that time (n = 6). 100% reported some improvement, 33.3% reported significant improvement. Improvements included average call quality (4/5), frequency of recent NEWS (3.7/5), and availability of Key clinical information (3.5/5).

Blank space feedback highlighted the tool's clarity and simplicity.

**Conclusion:**

This QI project was able to highlight and address a key issue in clinical care in a simple, and very low cost manner. Improvements were demonstrated after one month of intervention, and a more in-depth trust-wide rollout of the project is being discussed.

